# Molecular typing of a *Legionella pneumophila* outbreak in Ontario, Canada

**DOI:** 10.1099/jmm.0.46738-0

**Published:** 2007-03

**Authors:** Matthew W. Gilmour, Kathryn Bernard, Dobryan M. Tracz, Adam B. Olson, Cindi R. Corbett, Tamara Burdz, Betty Ng, Deborah Wiebe, George Broukhanski, Peter Boleszczuk, Patrick Tang, Frances Jamieson, Gary Van Domselaar, Francis A. Plummer, Jody D. Berry

**Affiliations:** 1 National Microbiology Laboratory, Public Health Agency of Canada, 1015 Arlington Street, Winnipeg, Manitoba R3E 3R2, Canada; 2 Department of Medical Microbiology and Infectious Diseases, University of Manitoba, Winnipeg, Manitoba, Canada; 3 Central Public Health Laboratory, Ministry of Health and Long Term Care, Toronto, Ontario, Canada

## Abstract

An outbreak of Legionnaires' disease at a long-term care facility in Ontario, Canada from September to October 2005 resulted in the death of 23 residents and the illness of 112 other people. In response, molecular methods were developed to detect *Legionella pneumophila* in clinical lung samples and to subtype isolates from clinical and environmental samples. The targeted genetic loci included *Legionella*-specific virulence determinants (*mip*, *icmO*, *sidA* and *lidA*) and core bacterial determinants (*ftsZ*, *trpS* and *dnaX*). An established amplified fragment length polymorphism typing method provided the first indication of genetic relatedness between strains recovered from clinical samples and strains cultured from environmental samples taken from the outbreak site. These associations were verified using the European Working Group for *Legionella* Infections sequence-based typing protocol targeting the *flaA*, *pilE*, *asd*, *mip*, *mompS* and *proA* loci. These molecular typing methods confirmed the outbreak source as a contaminated air conditioning cooling tower.

## INTRODUCTION

*Legionella pneumophila* is a ubiquitous bacterium in natural aquatic environments that can also persist in human-controlled systems containing water, such as air conditioning and plumbing infrastructures. Intracellular growth in protozoa can permit this pathogen to survive chlorination, and the generation of aerosols in these systems contributes to the transmission to humans where infection of alveolar macrophages results in respiratory illness (Steinert *et al.*, 2002; McDade *et al.*, 1977). The first recognized outbreak of Legionnaires' disease occurred in 1976 in Philadelphia, USA, and *Legionella* is now accepted as a significant cause of community- and nosocomial-acquired pneumonia, including in long-term care facilities (Seenivasan *et al.*, 2005).

During outbreaks of Legionnaires' disease the recovery or detection of *L. pneumophila* in clinical specimens can be sufficient for detection of the aetiological agent. However, due to the ubiquitous nature of this bacterium in the environment, molecular typing methods are needed both to determine the relatedness of outbreak strains and for definitive identification of the outbreak source. Culture of clinical specimens (e.g. from the lower respiratory tract) is the gold standard for diagnosis of Legionnaires' disease but rapid detection of *L. pneumophila* serogroup 1, the most common subtype associated with human disease, can be achieved with immunofluorescence and serological assays targeting *L. pneumophila* antigen and *L. pneumophila*-specific antibodies, respectively (Fields *et al.*, 2002). Molecular detection of genus and species is through assays targeting characteristic sites in the rRNA and *mip* genes, respectively (Fields *et al.*, 2002; Ballard *et al.*, 2000; Wilson *et al.*, 2003; Rantakokko-Jalava & Jalava, 2001). The *mip* locus is also one of six loci used in a sequence-based typing (SBT) scheme for the molecular subtyping of *L. pneumophila* strains (Gaia *et al.*, 2005). The *mip* locus therefore encodes conserved species-specific polymorphisms useful for molecular diagnostics and also strain-specific polymorphisms that can distinguish amongst *L. pneumophila* isolates. The effectiveness of the SBT scheme for determining the genetic relatedness of *L. pneumophila* outbreak-associated strains, in comparison to other subtyping methods such as PFGE, has been previously demonstrated (Scaturro *et al.*, 2005). In this study, molecular reagents were developed during an outbreak of Legionnaires' disease for the detection of *L. pneumophila* in clinical specimens and typing of clinical and environmental isolates.

## METHODS

The case definition of Legionnaires' disease was chest X-ray confirmed pneumonia, clinical diagnosis or pathological evidence of pneumonia at autopsy, and living or working within 3 kilometres (1.9 miles) of the outbreak site since a month prior to the outbreak. Each case of Legionnaires' disease was definitively confirmed after a positive culture for *L. pneumophila* serogroup 1, a positive urine antigen test (Binax) or positive immunofluorescence antibody serology (standing titre of ⩾1 : 256 or fourfold seroconversion of ⩾1 : 128).

*Legionella* strains were recovered from clinical specimens and environmental samples as described by Wilkinson (1988) and Fields (1994). Briefly, *Legionella* spp. from environmental samples were first selected using buffered charcoal yeast agar (BCYE) plates supplemented with polymyxin B, anisomycin and vancomycin (PAV). Colonies resembling *Legionella* spp. on the BCYE-PAV plates were then differentiated using BCYE, BCYE (lacking cysteine) and blood agar plates. Isolation of *Legionella* spp. from lung samples was performed using solely BCYE plates. All incubations were performed at 37 °C in candle jars or CO_2_ incubators. Lung autopsy materials were transported in viral transport media, swabs and water samples were collected in sterile containers, and air samples were collected with a XMX-CV aerosol collection system (Dycor). Direct fluorescent antibody assay (DFA) analysis on clinical specimens were done using monoclonal or polyclonal DFA products directed towards *L. pneumophila* serogroups (Prolab and Monofluo *L. pneumophila* kit; Bio-Rad). Additionally, total DNA was prepared from 19 lung autopsy samples from 7 patients using the DNeasy tissue extraction kit (Qiagen).

Oligonucleotides designed for molecular detection and typing are described in Table 1. PCR was performed using these oligonucleotides (1 μM), dNTPs (0.2 mM), magnesium sulfate (2 mM), Tris/sulfate (60 mM; pH 8.9), ammonium sulfate (18 mM) and 0.5 U High Fidelity Platinum *Taq* polymerase (Invitrogen) per 25 ml reaction, and the following thermocycling conditions: 94 °C for 5 min; 35 cycles of 94 °C for 30 s, 55 °C for 30 s and 68 °C for 30 s (except with *sidA*, *lidA* and *icmO* 1 min); and a one-round final extension at 68 °C for 5 min. DNA sequencing was performed with the oligonucleotides used to generate the amplicon. SBT using targets designed by the European Working Group for *Legionella* Infections (EWGLI) and amplified fragment length polymorphism (AFLP) analysis were performed as described by Gaia *et al*. (2003, 2005) and Valsangiacomo *et al*. (1995).

## RESULTS AND DISCUSSION

An outbreak of Legionnaires' disease at an extended care facility in Scarborough, Ontario, Canada, from September to October, 2005 resulted in 23 deaths. All mortalities occurred with residents of the care facility (hereafter, ‘outbreak site’) and additional cases were identified in 47 residents, 39 staff and 21 visitors to the outbreak site, as well as in 5 occupants and staff of a neighbouring care facility and residence (hereafter, ‘adjacent site’). A total of 82 people were affected with symptoms resembling Legionnaires' disease and 53 people presented with the milder form of illness cause by *Legionella*, Pontiac fever. A total of 79 people were hospitalized, of which 54 were residents at the outbreak site. This study describes the molecular detection of *L. pneumophila* in the lung autopsy material from the first mortalities, and the subtyping of *L. pneumophila* strains subsequently isolated from clinical and environmental samples.

Molecular detection of *L. pneumophila* in lung tissue DNA extractions and subtyping of pure cultures was achieved using species-specific oligonucleotides (Table 1). These were designed for various genetic loci after comparative analyses of the complete sequence data from three *L. pneumophila* group 1 strains (Lens, Paris and Philadelphia: GenBank accession numbers NC_006369, NC_006368 and NC_002942, respectively) and with the other publicly available bacterial sequences. Oligonucleotides corresponded to conserved regions amongst the three *L. pneumophila* genomes, but were unique compared to other genera and/or species (data not shown). The target loci included *Legionella*-specific virulence determinants (*mip*, *icmO*, *sidA* and *lidA*) and core bacterial determinants (*ftsZ*, *trpS* and *dnaX*). These core bacterial determinants have been predicted to contain species-specific polymorphisms (Zeigler, 2003), and we have previously utilized *ftsZ* and *trpS* for speciation of *Vibrio* spp. and detection of *Salmonella enterica* serovar Typhi, respectively (Tracz *et al.*, 2006, 2007). Published primer sets specific for the *Legionella* genus (16S rRNA) and human *β*-globin locus were used as additional control reactions (Raggam *et al.*, 2005; Keller & Mannack, 1993).

PCR-based detection assays for *L. pneumophila* were conducted on 19 lung tissue DNA extractions (from 7 different patients) using *ftsZ*, *trpS*, *dnaX* and *mip L. pneumophila*-specific primer sets; control reactions included 293T tissue culture cells spiked with *Legionella micdadei* ATCC 33218 or spiked with *L. pneumophila* ATCC 33153 (Table 2). Using this approach, all clinical lung samples were positive for the presence of *L. pneumophila*, except for a single lung tissue extract (05-L-025, patient 3). The two other lung specimens from patient 3 were positive for *L. pneumophila* and the control PCR reactions for specimen 05-L-025 failed, indicating that DNA extraction was likely unsuccessful for this individual specimen. DFA testing on one lung autopsy specimen from each patient was positive for *L. pneumophila* serogroup 1, whereas *L. pneumophila* was cultured from lung specimens for six of the seven patients.

Environmental sampling was performed throughout the outbreak site and the adjacent site, including cooling towers, air filtration systems and internal plumbing systems. Samples included sediments and biofilms in these infrastructure, collection of standing and free-flowing water, and collection of air-borne particulate matter. Additionally, sampling was performed of the soil and natural water systems in the surrounding area, which included a ravine and creek directly behind the outbreak site. Notably, PCR-based detection of *L. pneumophila* directly in environmental samples failed, including those that ultimately were cultured for this organism. The amount of *L. pneumophila* DNA extracted from these samples was likely below the detection threshold for these methods, and therefore these methods might be limited to analysis of pure culture and clinical samples. The only environmental samples that yielded *L. pneumophila* isolates were associated with the cooling towers at both the outbreak and adjacent sites (Table 3). Isolates 05-L-77, -79, -192 and -193 were typed as serogroup 1, corresponding to the same serogroup observed in the lung autopsy material by DFA analysis.

**Fig. 1. f1:**
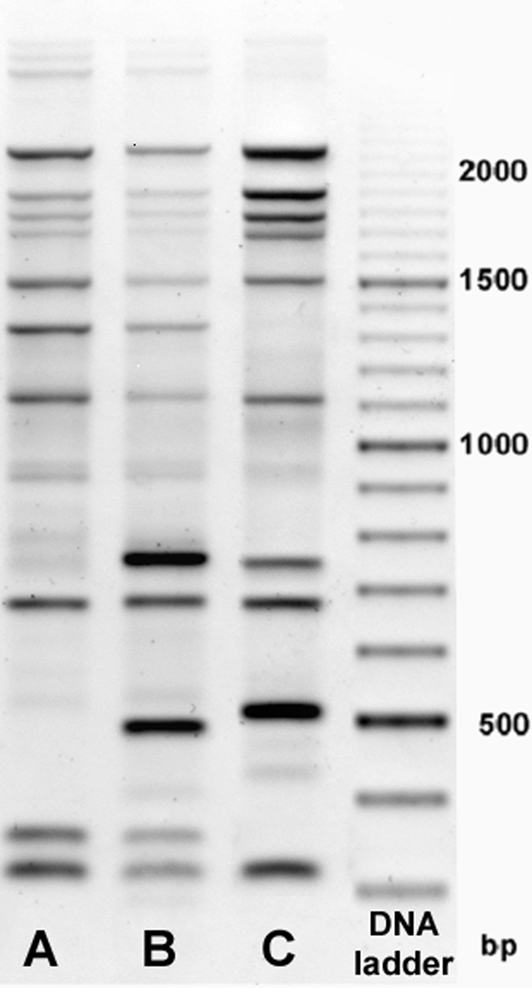
AFLP analysis of outbreak-associated (A, B) and adjacent site (C) isolates. Molecular mass markers are indicated.

To discriminate the environmental isolates beyond the level of serogroup, and to determine genetic relatedness to the lung isolates, AFLP analysis was used as a rapid molecular typing method (Fig. 1). The AFLP pattern produced from adjacent site isolates was different than the patterns obtained for both the outbreak site cooling tower and lung isolates, wherein two similar (but non-identical) patterns were observed amongst both groups of these outbreak-associated isolates. These data provided the first indication that the cooling tower at the outbreak site was responsible for the event.

**Fig. 2. f2:**
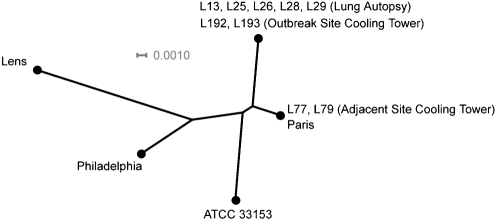
Genetic relatedness of outbreak, environmental and reference *L. pneumophila* strains. Phylogeny is based upon a neighbour-joining tree of the concatenated segments of core bacterial loci (*trpS*, *ftsZ* and *trpS*) and *Legionella*-specific virulence determinants (*lidA*, *icmO*, *sidA*, total 4243 nucleotides per entry). GenBank accession numbers for previously sequenced loci are presented in the text, and strains from the current study are abbreviated from the form 05-L-0XX to LXX. The source is indicated in parentheses. Bar, scale of the distance score.

The clinical and environmental *L. pneumophila* isolates were also prepared as template for DNA sequencing using the *ftsZ*, *trpS*, *dnaX*, *icmO*, *sidA* and *lidA* loci. Phylogenetic comparisons indicated that each of the lung isolates and strains recovered from the cooling tower at the outbreak site had identical genotypes at all loci (Fig. 2). Furthermore, the outbreak site and lung isolates had a different genotype at all loci in comparison to reference strains and strains isolated at the adjacent site, whereas the adjacent site isolates had identical genotypes as the endemic ‘Paris' strain. SBT using an established protocol (Gaia *et al.*, 2005, 2003) was also utilized to determine the genetic relatedness of the isolates (Table 3), and the allelic profile of the lung-associated strains was again identical to the strains recovered from the outbreak site cooling tower, but different from the profile of isolates from the adjacent site. Notably, none of the individual SBT alleles reported in this study were novel to the database maintained by EWGLI, but the cumulative profile of the outbreak strain had not been reported before and therefore no associations to previously examined strains were evident. However, the isolates from the cooling tower of the adjacent site encoded the most frequently recorded profile in the EWGLI database (N. Fry, personal communication).

Molecular reagents were developed during this outbreak of Legionnaires' disease to supplement bacterial culture and immunological detection methods for *L. pneumophila* in clinical lung specimens. Additional validation will be required to confirm the specificity and sensitivity of the primer sets in other clinical samples such as bronchial alveolar lavage fluid and urine. Molecular typing analyses (both SBT and AFLP) demonstrated that isolates from the cooling tower at the outbreak site were related to isolates from lung autopsy specimens, but divergent from isolates recovered from an adjacent building and reference strains. Notably, AFLP analyses distinguished two clones amongst the human and environmental outbreak-associated isolates suggesting increased sensitivity for this method; however, the nature of the observed genetic diversity was undefined. The genetic loci developed in this study for SBT effectively determined the relatedness of clinical and environmental samples, but the established EWGLI SBT protocol was of increased utility because it similarly differentiated the *L. pneumophila* strains but additionally allowed for comparison to a global collection of SBT data deposited in a web-accessible database.

## Figures and Tables

**Table 1. t1:** Oligonucleotides developed in this study for the detection and subtyping of *L. pneumophila*

**Target**	**Oligonucleotide**	**Sequence (5′ to 3′)**	**Product size (bp)**	Purpose*
*dnaX*	GIL296	ATACCTGTGTTGCCATTGAGC	255	PCR-Lpn ID
	GIL297	ATTTTTGTGGGTCAGTCGTTG		PCR-Lpn ID
*dnaX*	GIL313	TTTGCGCTGGTGTATTATCATC	936 (with GIL296)	PCR-sequencing
*ftsZ*	GIL298	GCGATTGCATCTCCTTTATTG	351	PCR-Lpn ID
	GIL299	CTGAGCCTGCTTACGAATCAC		PCR-Lpn ID
*ftsZ*	GIL312	CGATGCACAAGCTTTAAGAGG	946 (with GIL299)	PCR-sequencing
*trpS*	GIL300	TCAGCCTAATTTGTCGATTGG	274	PCR-Lpn ID
	GIL301	TTGCCAAACAGGACATTTTTC		PCR-Lpn ID
*trpS*	GIL308	TTGATTGGCTAGCTTGTGGAG	999	PCR-sequencing
	GIL309	AATCAAGCCCCATGACTTCTC		PCR-sequencing
*mip*	GIL306	GCTTTAACCGAACAGCAAATG	267	PCR-Lpn ID
	GIL307	AACGGTACCATCAATCAGACG		PCR-Lpn ID
*lidA*	GIL314	TCACATCAAGTTAAAACATCAG	882	PCR-Lpn ID
	GIL315	ATGCTCACGCTGTAAGGATTG		PCR-Lpn ID
*icmO*	GIL316	AATTTTCGGTTCAACGGGTAG	763	PCR-Lpn ID
	GIL317	CAGTGCGGGTAATAAAACCAC		PCR-Lpn ID
*sidA*	GIL318	GAAATTCGTGCCATAGAACTCC	330	PCR-Lpn ID
	GIL319	CGGGGCTATGATTTCTTCTATG		PCR-Lpn ID

*Lpn ID, *L. pneumophila*-specific detection.

**Table 2. t2:** PCR-based detection of *L. pneumophila* Templates included clinical lung autopsy material and tissue culture spiked with live bacterial culture (prepared using a Qiagen tissue extraction kit) or pure bacterial culture (prepared from boiled cell lysates).

Sample*	**Patient**	**Description**	***dnaX***	***ftsZ***	***trpS***	***mip***	**16S†**	***β*-globin‡**
*L. pneumophila* ATCC 33153	na	Pure culture	+	+	+	+	+	−
*L. micdadei* ATCC 33218	na	Pure culture	−	−	−	−	+	−
293T tissue culture	na	Tissue culture	−	−	−	−	−	+
293T+*L. pneumophila*	na	Spiked tissue	+	+	+	+	+	+
293T+*L. micdadei*	na	Spiked tissue	−	−	−	−	+	+
05-L-011	1	Autopsy tissue	+	+	+	+	+	+
05-L-012 (DFA+)	1	Autopsy tissue	+	+	+	+	+	+
05-L-013	1	Autopsy tissue	+	+	+	+	+	+
05-L-014	2	Autopsy tissue	+	+	+	+	+	+
05-L-015	2	Autopsy tissue	+	+	+	+	+	+
05-L-024 (DFA+)	2	Autopsy tissue	+	+	+	+	+	+
05-L-016	3	Autopsy tissue	+	+	+	+	+	+
05-L-017	3	Autopsy tissue	+	+	+	+	+	+
05-L-025 (DFA+)	3	Autopsy tissue	−	−	−	−	−	−
05-L-018	4	Autopsy tissue	+	+	+	+	+	+
05-L-019	4	Autopsy tissue	+	+	+	+	+	+
05-L-027 (DFA+)	4	Autopsy tissue	+	+	+	+	+	+
05-L-020	5	Autopsy tissue	+	+	+	+	+	+
05-L-021	5	Autopsy tissue	+	+	+	+	+	+
05-L-026 (DFA+)	5	Autopsy tissue	+	+	+	+	+	+
05-L-022	6	Autopsy tissue	+	+	+	+	+	+
05-L-023	6	Autopsy tissue	+	+	+	+	+	+
05-L-028 (DFA+)	6	Autopsy tissue	+	+	+	+	+	+
05-L-029 (DFA+)	7	Autopsy tissue	+	+	+	+	+	+

na, Not applicable. *DFA+, DFA detected *L. pneumophila* serogroup 1 in the clinical specimen. †*Legionella* genus-specific primers (Raggam *et al.*, 2005). ‡Human-specific primers (Keller & Mannack, 1993).

**Table 3. t3:** SBT of *L. pneumophila* isolates cultured from lung autopsy tissue or from environmental samples The allele profile number for each locus is indicated. AFLP analysis was performed on selected isolates and the patterns correlated to those illustrated in Fig. 1.

**Strain**	**Site of collection; AFLP result**	***flaA***	***pilE***	***asd***	***mip***	***mompS***	***proA***
05-L-013	Lung, patient 1	2	19	5	10	18	1
05-L-025	Lung, patient 3	2	19	5	10	18	1
05-L-026	Lung, patient 5	2	19	5	10	18	1
05-L-028	Lung, patient 6	2	19	5	10	18	1
05-L-029	Lung, patient 7	2	19	5	10	18	1
060017	Lung, patient 4; pattern A	2	19	5	10	18	1
060018	Lung, patient 1; pattern B	2	19	5	10	18	1
060019	Outbreak site, cooling tower; pattern A	2	19	5	10	18	1
060020	Outbreak site, cooling tower middle cavity; pattern B	2	19	5	10	18	1
05-L-0192-1	Outbreak site, cooling tower bottom cavity	2	19	5	10	18	1
05-L-0192-2	Outbreak site, cooling tower bottom cavity	2	19	5	10	18	1
05-L-0192-3	Outbreak site, cooling tower bottom cavity	2	19	5	10	18	1
05-L-0192-4	Outbreak site, cooling tower bottom cavity	2	19	5	10	18	1
05-L-0192-5	Outbreak site, cooling tower bottom cavity	2	19	5	10	18	1
05-L-0192-6	Outbreak site, cooling tower bottom cavity	2	19	5	10	18	1
05-L-0192-7	Outbreak site, cooling tower bottom cavity	2	19	5	10	18	1
05-L-0193	Outbreak site, cooling tower	2	19	5	10	18	1
CPHL-3	Adjacent site; pattern C	1	4	3	1	1	1
CPHL-5	Adjacent site; pattern C	1	4	3	1	1	1
CPHL-7	Adjacent site; pattern C	1	4	3	1	1	1
05-L-077-1	Adjacent site, west cooling tower	1	4	3	1	1	1
05-L-079-2	Adjacent site, east cooling tower	1	4	3	1	1	1
ATCC 33153	*L. pneumophila* group 1 (Knoxville) reference strain	7	6	17	3	24	11

## Abbreviations

- AFLP, amplified fragment length polymorphism
- DFA, direct fluorescent antibody assay
- EWGLI, European Working Group for *Legionella* Infections
- SBT, sequence-based typing
